# Effectiveness of Benson’s relaxation and Mitchell’s relaxation along with conventional physiotherapy exercise on pain, urinary incontinence, sleep quality, and activities of daily living in lower segmental cesarean section: a comparative study

**DOI:** 10.1186/s13063-026-09761-w

**Published:** 2026-05-15

**Authors:** Neha Krishnakumar Yadav, Ravi Lashkari, Shruti Deshpande

**Affiliations:** Department of Community Health Physiotherapy, Ravi Nair Physiotherapy College, Datta Meghe Institute of Higher Education and Research, Sawangi (Meghe), Wardha, Maharashtra India

**Keywords:** LSCS, Benson’s relaxation technique, Mitchell’s relaxation technique, Pain, Urinary incontinence, Sleep quality, Activities of daily living

## Abstract

**Background:**

Therapeutic exercises that help reduce stress and anxiety, both mentally and physically, are called relaxation techniques. In order to ascertain the benefits of the two relaxation techniques on pain, urine incontinence, sleep quality, and activities of daily living, this study will compare them with the conventional physiotherapy exercise in lower segmental cesarean sections (LSCS).

**Methods:**

Forty-six LSCS subjects with pain, urine incontinence, sleep quality, and activities of daily life will participate in a comparative study that is a blinded (statistical) analysis. The participants will be divided into two groups following randomization: (1) Benson’s relaxation and (2) Mitchell’s relaxation, and both groups will receive exercises from conventional physiotherapy. Every group will go through the initial evaluation and a post-intervention assessment.

**Discussion:**

This study is anticipated to improve pain, urinary incontinence, sleep quality, and activities of daily living following a lower segmental cesarean section. Following the completion of the study, the data will be published. If the benefits are demonstrated, Benson’s or Mitchell’s relaxation approach is used in conjunction with conventional physiotherapy exercises after lower-segmental cesarean sections.

**Result:**

The protocol would be given for 4 weeks. The initial assessment would be conducted before the intervention, and the post-assessment would be performed after the completion of the 4 weeks of intervention.

**Trial registration:**

Trial REF/2025/03/102462, Registration number- CTRI/2025/04/085318. Date of registration-22/04/2025.

## Administrative information

**Table:** 

Title {1}	Effectiveness Of Benson’s Relaxation and Mitchell’s Relaxation Along with Conventional Physiotherapy Exercise on Pain, Urinary Incontinence, Sleep Quality and Activities of Daily Living in Lower Segmental Cesarean Section: A Comparative Study
Trial registration {2a and 2b}	Trial- REF/2025/03/102462, Registration number- CTRI/2025/04/085318 Date of registration- 22/04/2025
Protocol version {3}	-
Funding {4}	No Funding
Author details {5a}	1st—Dr Neha Krishnakumar Yadav, Junior Resident, Department of Community Health Physiotherapy, DMIHER 2nd—Dr Ravi Lashkari, Associate Professor, Department of Community Health Physiotherapy, DMIHER 3rd – Dr Shruti Deshpande, Assistant Professor, Department of Community Health Physiotherapy, DMIHER
Name and contact information for the trial sponsor {5b}	Not Applicable
Role of sponsor {5c}	-

## Introduction

### Background and rationale {6a}

The rate of cesarean sections has dramatically increased globally in recent years. Two types of cesarean section are performed: elective and emergency cesarean section [1]. Women undergo a number of physiological, psychological, and social changes during the postpartum phase [2]. These changes result in a number of issues that impact women’s daily lives. Following a cesarean section, women seem to be more susceptible to sleep disturbances. This discrepancy is particularly noticeable in late pregnancy and the early postpartum period, which has been identified as a time of vulnerability to sleep problems [3]. A loss of bladder control known as urinary incontinence can range from a minor leak of pee following coughing, sneezing, or laughing to an absolute inability to control urination. Urinary incontinence is most common in women during pregnancy and after giving birth [4]. One of the most prevalent issues in the postpartum phase is back discomfort. Seven out of ten women who give birth have back pain, which is more than 70% of cases, according to statistical evidence. Obesity, poor posture, sitting, walking, and standing are all associated with postpartum back pain. According to the study’s findings, post-anesthesia back discomfort can be reduced by practicing posture correction, yoga, meditation, lumbar support, rest, and massage [5].

Therapeutic exercises that help reduce stress and anxiety both mentally and physically are known as relaxation techniques [6]. Relaxation techniques can also be used as adjunctive therapies in a range of healthcare settings to treat patients with a variety of diseases, mostly but not exclusively stress, anxiety, depression, and pain [7]. Psychophysiological therapies such as relaxation techniques reduce stress by encouraging mental and physical relaxation [8]. These methods effectively lessen pain and anxiety and are frequently used in physiotherapy [9]. A clear description of therapeutic relaxation appears challenging, even though relaxation is a widely recognized and frequently utilized term [10].

Mitchell’s method is a silent, solitary aural relaxing technique. In addition to including deep breathing, relaxation techniques, and guided imagery, it highlights the mind–body psychoneuroimmunological relationship and suggests total involvement and autonomy. It is theorized that the posture associated with stress causes the neurological and endocrine systems to change, increases muscle tension, and encourages muscular stiffness and dystonic patterns [11].

Benson’s relaxation technique was developed by Herbert Benson in 1970 as a straightforward method of stress relief [12]. The quality of sleep, general quality of life, and pain intensity all improved as a result of this tactic. At the same time, it promoted physical activity and reduced mood disorders and anxiety [13]. Crucially, Benson’s relaxation method has no negative effects on people, offers a number of advantages, and is easy to apply [14].

## Objectives {7}

The study aims to compare Benson’s relaxation (group A) and Mitchell’s relaxation (group B) along with conventional physiotherapy exercises in both groups to evaluate the pain, urinary incontinence, improving the sleep quality and activities of daily living in LSCS subjects with the help of outcomes to determine which group has better improvement.

## Trial design {8}

It will be a quantitative, analytical, comparative study with a single-blinded study design, meaning that participants will be blinded. We conducted this protocol in accordance with SPIRIT (Standard Protocol Items: Recommendation for Interventional Trials).

Before randomization, consent forms will be used to get written informed consent from each participant.

## Methods: participants, interventions, and outcomes

### Study setting {9}

The study will be carried out at the Acharya Vinoba Bhave Rural Hospital’s Obstetrics and Gynaecology Ward at the Datta Meghe Institute of Medical Sciences in Sawangi (Meghe), Wardha, Maharashtra, India. The Obstetrics and Gynaecology ward’s patients will undergo screening to determine their eligibility.

### Eligibility criteria {10}

The inclusion criteria consist:

1. Women from 20 to 40 years of age,
2. Type of LSCS: emergency and elective,
3. Individuals with any mode of conception,
4. Primigravida or Multigravida,
5. Individuals with written consent.

The exclusion criteria:

1. Non-co-operative patients,
2. History of chronic back pain unrelated to pregnancy,
3. Vaginal delivery,
4. Individuals with sleep disorders,
5. Individuals on sleep medications (hypnotics),
6. Cognitive impairment.

### Who will take informed consent? {26a}

The principal investigator (PI) will take the informed consent.

### Additional consent provisions for collection and use of participant data and biological specimens {26b}

Not applicable.

#### Rationale

The principal investigator will collect consent from the subjects; no additional consent is needed. This protocol does not involve biological specimens as part of the process.

## Interventions

### Explanation for the choice of comparators {6b}

Both Benson’s and Mitchell’s relaxation techniques are psychophysiological treatments that have been shown to lower stress, pain, and anxiety and also enhance sleep quality and overall health. However, there is a lack of comparison between these two techniques in LSCS subjects. Both techniques are non-pharmacological, easy, and safe to implement in LSCS subjects without any adverse effects.

### Intervention description {11a}

#### Conventional physiotherapy exercise

Patients who meet the inclusion criteria will be selected following ethical clearance from the Institutional Ethical Committee.

Pretreatment and post-treatment data will be recorded with the help of outcome measures. After dividing patients into two groups, each group A and B will receive Mitchell’s relaxation and Benson’s relaxation, respectively, for 30 min along with breathing exercises; followed by this, patients will receive interventions 5 days a week. This relaxation would be given with conventional physiotherapy exercises in both groups. Intervention will be given on POD-2 after LSCS in both the groups A and group B.

**Table:** 

Sr no.	Intervention	Procedure
1	Thoracic expansion exercise	Larger than normal breaths are taken during inspiration, along with shoulder are expanded in flexion and during expiration shoulder are brought back to the neutral position
2	Kegel’s exercise	Tighten the pelvic floor muscle as you are holding the urine, maintain the contraction for 5 s, and then relax
3	Isometrics hamstrings	Bend the knee while lying supine in the bed, press the heel on the bed, and feel the tightness on the back of your thigh
4	Isometrics quadriceps	Press the knees on the bed, and the heel should raise the bed a little. Hold the contraction for 5 s and then relax
5	Ambulation	Walk with the proper abdominal binder to provide for the stitches

##### Group A

Subjects will receive 30 min Mitchell’s relaxation exercise per day, along with conventional physiotherapy exercises in both groups. Follow-up of the patient will be done daily. Diaphragmatic breathing exercises and a series of serial isotonic contractions aimed at reciprocal inhibition are both components of Mitchell’s physiological relaxation technique.

##### Group B

Benson’s relaxation will be administered to LSCS sufferers for 30 min. It is divided into five sections: (1) a calm environment: picking a spot devoid of disturbances; (2) it is crucial to assume a calming position, such as standing, sitting, sleeping, or walking; (3) appropriate acceptance: making sure that every muscle is relaxed, starting from the soles of the feet and working your way up to the face muscles; (4) concentration: maintaining awareness of breathing patterns and using the nose for inspiration and the mouth for expiration; (5) appropriate acceptance: keeping a calm demeanor. The techniques work by decreasing endogenous catecholamine levels and the activity of the sympathetic nervous system.

### Criteria for discontinuing or modifying allocated interventions {11b}

At any time, participants may request to stop or alter the intervention they have been assigned, or the investigator may decide that they should stop or alter the intervention if they suffer any discomfort, an adverse event, or a worsening of their condition.

### Strategies to improve adherence to interventions {11c}

1. Education and demonstration of participants: At the beginning of the study, participants will get explicit written and verbal instructions along with examples of the designated physiotherapy exercises and relaxation techniques. This guarantees that participants comprehend the processes and their significance.
2. Supervised sessions: To guarantee proper technique and promote participation, interventions will be given or overseen by a principal investigator while the participants are in the hospital.

### Relevant concomitant care permitted or prohibited during the trial {11d}

#### Permitted concomitant care

1. Standard LSCS nursing care according to the hospital.
2. Conventional physiotherapy exercise according to the protocol in both groups.
3. Regular medications that were prescribed to postpartum women.

#### Prohibited concomitant care

1. Any other relaxation techniques.
2. Yoga, meditations, or physiotherapeutic modalities.
3. Use of hypnotics.

### Provisions for post-trial care {30}

Not applicable.

#### Rationale

Both relaxation techniques are safe and non-invasive without adverse effects.

### Outcomes {12}

#### Primary outcomes

1. Visual analogue scale (VAS) is one instrument used to rate pain. It is a simple and widely used method in medicine to assess the degree of discomfort.
2. Incontinence severity index (ISI) is a questionnaire that measures the severity of urinary incontinence. It is used to categorize incontinence as slight, moderate, severe, or very severe.

#### Secondary outcomes

1. Pittsburgh Sleep Quality Index (PSQI) evaluates that the sleep quality is the goal. Respondents are asked how often they experienced particular sleep problems in addition to rating the quality of their sleep.
2. Barthel Index Scale was used to evaluate scale independence. The overall score is between zero and one hundred points. Functional independence increases with a higher score [15].

### Participant timeline {13}

**Table:**
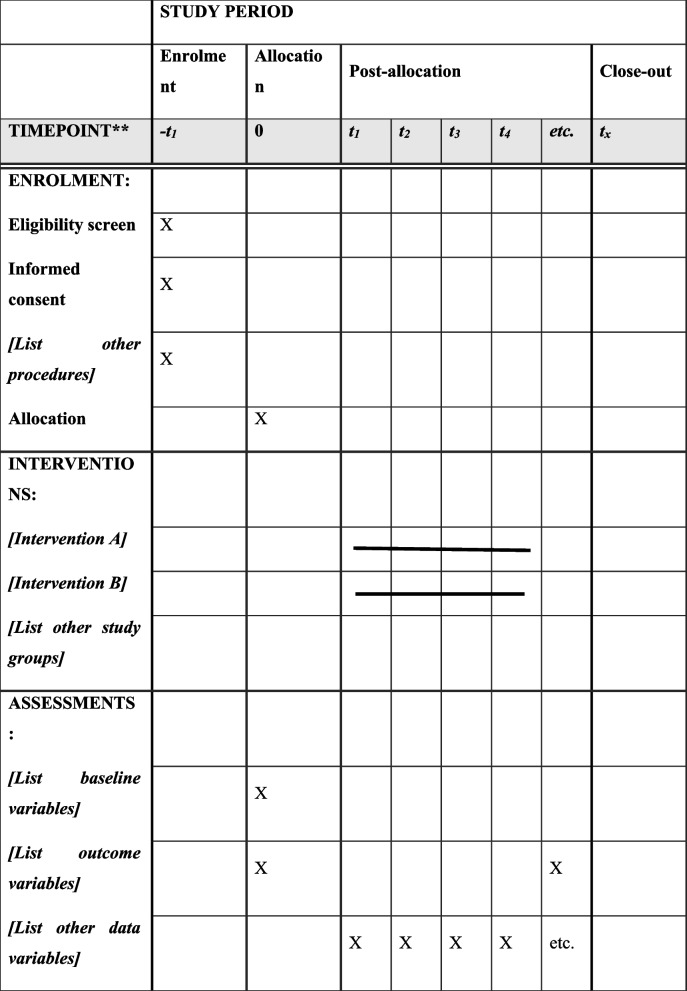


^**^Statistical significant

### Sample size {14}

#### Sample size: calculation for comparing two means at pain severity using VAS

##### Formula used

The formula for calculating the sample size for comparing two means is:

$$n\geq\left[\left(Z\_\left\{1-\alpha/2\right\}+Z\_\left\{1-\beta\right\}\right)^2\cdot\left(\upsigma_1{}^2+\upsigma_2{}^{2/r}\right)\right]/\left(\mu_2-\mu_1\right)^2$$

where:

- *Z*_{1-*α*/2} is the *Z*-score corresponding to the two-sided significance level.
- *Z*_{1-*β*} is the *Z*-score corresponding to the desired power.
- σ_1_^2^ and σ_2_^2^ are the variances of the two groups.
- *r* is the ratio of the group sizes.
- (*μ*_2_—*μ*_1_) is the difference in means between the two groups.
- Alpha (*α*): 0.05 (significance level) = 1.96
- Beta (*β*): 0.2 (power = 1—*β* = 0.8) = 0.084
- Mean in group 1 (*μ*_1_): 7.916 (A s per reference article)
- Standard deviation in group 1 (σ_1_): 2.256
- Mean in group 2 (*μ*_2_): 5.983
- Standard deviation in group 2 (σ_2_): 2.327
- Ratio of group sizes (group 2/group 1): 1:1

##### Results

- Minimum sample size needed for group 1: 23
- Minimum sample size needed for group 2: 23
- Total sample size required: 46

### Recruitment {15}

Clinical trial recruitment involves the screening of patients in Acharya Vinoba Bhave Rural Hospital, Obstetrics and Gynaecology Ward, identifying eligible participants who fulfill the eligibility criteria (inclusion and exclusion criteria) and with written informed permission from willing participants.

## Assignment of interventions: allocation

### Sequence generation {16a}

This process involves the allocation of the patient based on randomly generated numbers. The patient is required to select an envelope containing numbers. The patient’s assignment to the type of intervention will depend on the number.

### Concealment mechanism {16b}

#### Basic random sampling

The study participants will be randomly assigned using computer-generated random numbers. Randomization is guaranteed through computer-generated random numbers placed in opaque envelopes, ensuring unpredictability and equal chance of group assignment; this method minimizes selection bias as the sequence is pre-determined and concealed. Basic random sampling via these computer-generated numbers further supports balanced allocation across the two groups.

### Implementation {16c}

A blind individual without any medical training will create the random number sequence. Participants will be enrolled and assigned to the interventions by the study’s primary investigator.

## Assignment of interventions: blinding

### Who will be blinded {17a}

Outcome accessor would be blinded to the intervention.

### Procedure for unblinding if needed {17b}

No unblinding is permissible to reduce the risk of bias.

## Data collection and management

### Plans for assessment and collection of outcomes {18a}

1. Baseline assessment: Before giving any intervention, data will be collected from subjects at the beginning of the first week.
2. Intervention period: During the 4 weeks of the intervention, subjects will get both the relaxation technique (Benson’s and Mitchell’s), along with the conventional physiotherapy exercises.
3. Post-intervention assessment: After the fourth week of intervention, right after the intervention period, data will be gathered once again.

### Plans to promote participant retention and complete follow-up {18b}

#### Daily follow-up

To keep subjects engaged and track adherence, they will be contacted every day throughout the intervention period.

#### Pre- and post-intervention assessments

Clear checkpoints for retention and result measurement are provided by the data collection at baseline (beginning of week 1) and at the conclusion of the intervention (end of week 4).

#### Regular intervention schedule

To promote routine and subject commitment, both groups receive conventional physiotherapy exercises and a 30-min relaxation technique 5 days a week.

### Data management {19}

The frequency percentage for qualitative data and the mean and standard deviation for quantitative data will be used to summarize the information acquired.

### Confidentiality {27}

Any information about the subjects participating in the study shall be maintained confidential. Any patient-related information will only be used with due permission from the subjects.

### Plans for collection, laboratory evaluation, and storage of biological specimens for genetic or molecular analysis in this trial/future use {33}

Not applicable.

#### Rationale

Using established questionnaires and scales, the methodology of this protocol only focuses on clinical and patient-reported outcomes, including pain, urinary incontinence, sleep quality, and activities of daily life.

## Statistical methods

### Statistical methods for primary and secondary outcomes {20a}

#### Characteristic statistics

Frequencies and percentages will be used to summarize qualitative data, such as demographic factors.

The mean ± standard deviation will be used to express quantitative data, including ratings from the visual analogue scale, incontinence severity index, Pittsburgh Sleep Quality Index, and Barthel Index Scale.

#### Comparisons within the group (before and after the intervention)

If the data are normally distributed, the paired *t*-test will be employed.

If the data are not normally distributed, the Wilcoxon signed-rank test will be applied.

#### Comparisons between groups (Mitchell’s vs. Benson’s relaxation)

For data that is regularly distributed, the independent *t*-test will be employed.

Mann–Whitney for data that is not typically distributed, the *U* test will be applied.

### Interim analyses {21b}

Not applicable.

#### Rationale

This protocol does not mention conducting any intermediate analyses during the trial. Data will be gathered at baseline (pre-intervention) and at the conclusion of the 4-week intervention period (post-intervention), according to the study design. All statistical analyses must be carried out following the conclusion of data collection.

### Methods for additional analyses (e.g., subgroup analyses) {20b}

Not applicable.

#### Rationale

According to this protocol, the procedure does not specify any plans or techniques for further studies, such as adjusted or subgroup analysis.

### Methods in analysis to handle protocol non-adherence and any statistical methods to handle missing data {20c}

Not applicable.

For subjects who finish the research, the protocol mainly concentrates on pre- and post-intervention analyses, and all analyses are designed for participants who finish the pre- and post-intervention questionnaires.

### Plans to give access to the full protocol, participant-level data, and statistical code {31c}

#### Full protocol

No clear strategy for public access outside of the criteria for publishing and trial registration.

#### Participant level data

No intention to share.

#### Statistics code

No intention to share.

## Oversight and monitoring

### Composition of the coordinating center and trial steering committee {5d}

#### Coordinating center

##### Location

The study is conducted in the Acharya Vinoba Bhave Rural Hospital’s Obstetrics and Gynaecology Ward at the Datta Meghe Institute of Medical Sciences in Sawangi (Meghe), Wardha, Maharashtra, India.

##### Role

Subject recruiting, eligibility screening, randomization, intervention delivery, data collection, and data administration.

#### Trial steering committee

The principal investigator is in charge of participant enrollment, randomization, assignment to intervention groups, and overall study conduct.

Physician oversees participant safety, handles unfavorable outcomes, and reports to the institutional ethics committee.

The Ethical Committee oversees ethics and examines reports of undesirable events.

### Composition of the data monitoring committee, its role, and reporting structure {21a}

#### Safety monitoring

Throughout the trial, the lead physician keeps an eye on, assesses, and handles any unintended consequences or adverse occurrences.

#### Reporting

The Institutional Ethical Committee is notified of any unfavorable occurrences for additional monitoring and, if required, action.

#### Ethical oversight

The Institutional Ethical Committee makes sure that the trial is carried out in accordance with ethical guidelines and that subject safety is preserved.

### Adverse event reporting and harms {22}

The physician in charge of evaluating and managing inadvertent and requested adverse events, as well as any other unanticipated results of trial interventions or trial conduct, shall be informed of every adverse event that takes place. The reports will also be sent to the ethical committee.

### Frequency and plans for auditing trial conduct {23}

Not applicable.

#### Rationale

Because of the non-invasive procedures, the study can be regarded as low risk and a small sample size, which lessens the need for official auditing.

### Plans for communicating important protocol amendments to relevant parties (e.g., trial participants, ethical committees) {25}

Before any significant protocol modifications (such as adjustments to eligibility requirements, research findings, or methods) will be quickly reported to the Institutional Ethics Committee for evaluation and approval, all presently enrolled participants will be notified of any changes that may impact their safety, rights, or willingness to participate, and their consent will be sought again if needed. Any major modifications will also be addressed in the trial registry item. The protocol version history will have a record of every modification.

## Dissemination plans {31a}

Any data collected during or after the study will only be used for academic and research-related purposes, culminating in a publication in a reputed journal.

## Discussion

In order to assess the efficacy of Benson’s and Mitchell’s relaxation techniques, each in conjunction with conventional physiotherapy, for women recovering from lower segmental cesarean sections (LSCS), the protocol offers a carefully planned, single-blind, randomized comparative clinical trial. This study’s justification stems from the high and increasing prevalence of LSCS worldwide, which is linked to serious postpartum problems such as pain, urinary incontinence, sleep issues, and diminished capacity to carry out everyday tasks. Comprehensive rehabilitation is necessary since these issues affect not only physical healing but also psychological and social well-being.

Both Mitchell’s and Benson’s relaxation methods are well-researched psychophysiological therapies that have been demonstrated to enhance sleep quality and lessen stress, pain, and anxiety in a variety of populations. Nevertheless, there is a dearth of direct comparison data about their efficacy in LSCS patients. In order to fill this gap, the procedure enlists 46 women between the ages of 20 and 40. They are then randomly randomized to receive either Benson’s or Mitchell’s relaxation technique in addition to conventional physiotherapy exercise. Five days a week, for 30 min per session, the interventions are given under supervision to guarantee proper technique and adherence. This strategy minimizes confounding variables while enabling a strong comparison.

The visual analogue scale (VAS) for pain, the incontinence severity index (ISI) for urine incontinence, the Pittsburgh Sleep Quality Index (PSQI) for sleep quality, and the Barthel Index for activities of daily living are among the extensive and validated outcome measures used in this study. These instruments will be employed both before and after the intervention, allowing for a comprehensive evaluation of the methods’ effects on important facets of postpartum recuperation. Careful attention is paid to ethical issues: all participants provide their informed consent, and precise inclusion and exclusion standards are set up to guarantee participant security and data integrity. Both procedures are safe and non-invasive, and the protocol permits stopping them if there is any discomfort or unfavorable outcome. Education, demonstration, and supervised sessions are some strategies to increase adherence, which are essential for guaranteeing consistent implementation of the interventions and trustworthy outcomes.

There are various reasons why the study is important. It begins by directly contrasting two popular but little-studied relaxation methods in a group of women who are at a heightened risk of postpartum problems. Second, the study mirrors actual clinical practice by combining these methods with traditional physiotherapy, and it may help shape evidence-based recommendations for LSCS recovery. Third, a randomized, single-blind methodology and verified outcome measures improve the findings’ generalizability and dependability. In order to improve pain management, urinary continence, sleep quality, and everyday functioning for women following LSCS surgery, the study may encourage the wider use of relaxation techniques in post-cesarean physiotherapy protocols if the results show a substantial advantage. By treating the psychological as well as the physical components of healing, this would constitute a significant advancement in postpartum treatment and eventually improve the quality of life for this patient population. The limitation of this study is that it effectively compares the two relaxation techniques; the absence of a third arm receiving conventional physiotherapy alone limits the ability to determine the absolute additive benefit of either relaxation technique over standard care.

## Data Availability

“Following the completion of the study, data will be published.” This indicates that the primary conclusions and summary results will be published.

## Abbreviations

LSCS: Lower segmental cesarean section
VAS: Visual analogue scale
ISI: Incontinence severity index
PSQI: Pittsburgh Sleep Quality Index
